# 

**Published:** 1908-05-23

**Authors:** 


BLOOMSBURY W C

---

### Death of Mr. C. H. Byers.

Mr. Charles Henry Byers, who had been Secretary of the Metropolitan Hospital, Kingsland Road, for twenty-one years, died on Sunday, May 10, at the age of forty-nine years. Mr. Byers had been in indifferent health for some time, and two years ago he had an operation performed upon him. He was well known in the world of hospital administration, and was very popular with the many medical men who have been at different times associated with the Metropolitan Hospital. He took the keenest interest in the affairs of that institution up to the very last, and was for many years in close personal contact with the medical staff. His loss will be felt by a wide circle of friends, and his abilities will be missed by the hospital which he served so well.

### BOOKS RECEIVED.

Adam and Charles Black.

"Home Nursing and Hygiene." By Florence Hufton-Windust.

"Cancer: Relief of Pain and Possible Cure." By Skene Keith, M.D., and George E. Keith, M.B.

The Scientific Press, Ltd.

"Hints and Aids on Monthly Nursing."

Baillière, Tindall, and Cox.

"Lectures on Babies." By Ralph Vincent, M.D.

A Memoir of the late Dr. Edmund Symes-Thompson has been prepared by his wife, and will be issued immediately by Mr. Elliot Stock. Lord Balfour of Burleigh is a contributor to the volume, which also contains a portrait of Dr. Symes-Thompson and other illustrations.

### A London Sleeping Sickness Bureau.

The Colonial Office announces that at the instance of the late Secretary of State for the Colonies, and with the co-operation of the Government of the Sudan and the Royal Society, his Majesty's Government have decided to establish at Burlington House a bureau for the collection and general distribution of information with regard to sleeping sickness. The bureau will be under the general control and direction of an honorary committee appointed by the Secretary of State for the Colonies, and composed of the following: Sir West Ridgeway (Chairman), Sir Patrick Manson, Sir Rubert Boyce, Dr. Rose Bradford, Colonel D. Bruce, Mr. Walrond Clarke, and Mr. H. J. Read, with Mr. R. Popham Lobb, of the Colonial Office, as secretary. The main function of the bureau will be to collect and arrange information from all sources regarding sleeping sickness, and to distribute it as widely and quickly as possible among those who are engaged in combating the disease. The publications of the bureau will consist of scientific publications intended for medical and scientific workers, and publications of a less technical character for the use of Government officials, missionaries, and others resident in those districts. A map of the whole of tropical Africa is to be prepared showing the distribution of the disease and of the different species of blood-sucking insects which are suspected of conveying it. The present director of the bureau is Dr. A. G. Bagshawe, of the Uganda Medical Staff.

### THE GRADUATES' MEDICAL SCHOOLS.

DIARY FOR WEEK MAY 25 TO 30.

**NORTH-EAST LONDON POST-GRADUATE COLLEGE, Prince of Wales's General Hospital Tottenham, N.**

**At 4.30 p.m.**

**May 26,** Dr. G. N. Meachen, **Selected Skin Cases.**

**May 28,** Dr. Basil Price, **Clinical Pathology.**

**THE POST-GRADUATE COLLEGE, West London Hospital, Hammersmith, W.**

**At 12 noon.**

**May 25,** Dr. Low, **Pathological Demonstration.**

**At 12.15 p.m.**

**May 27, 29,** Dr. Pritchard, **Practical Medicine.**

**At 5 p.m.**

**May 25,** Dr. Robinson. **Gynæcology.—II.**
**May 26,** Dr. Seymour Taylor, **Relapses in Typhoid Fever.**
**May 27,** Mr. Lloyd Williams, **When to Extract.**
**May 28,** Mr. Baldwin, **Practical Surgery.—II.**
**May 29,** Dr. Abraham, **Cases of Skin Disease.**

**NATIONAL HOSPITAL FOR THE PARALYSED AND EPILEPTIC, Queen Square, Bloomsbury, W.C.**

**At 3.30 p.m.**

**May 26,** Dr. J. Taylor, **Cerebral Diplegia.**
**May 29,** Sir V. Horsley, **Surgery of the Nervous System.**

**MOUNT VERNON HOSPITAL, Hampstead.**

**At 5 p.m.**

**May 28,** Dr J. E. Squire, **Cases in the Wards.**

**CHARING CROSS HOSPITAL, W.C.**

Post-Graduate Demonstrations.

**At 4 p.m.**

**May 28,** Dr. Fenton, **Medical.**

**LONDON SCHOOL OF CLINICAL MEDICINE, Seamen's Hospital, Greenwich, S.E.**

**At 3.15 p.m.**

**May 26,** Mr. Carless, **Hæmorrhoids and their Treatment.**

**At 3.30 p.m.**

**May 27,** Mr. Cargill, **The Treatment of Corneal Ulcers.**

**At 2.30 p.m.**

**May 28,** Dr. Rankin, **Exophthalmic Goitre.**

**MEDICAL GRADUATES' COLLEGE AND POLYCLINIC, Chenies Street, W.C.**

**At 5.15 p.m.**

**May 25,** Dr. James Collier, **Myasthenia Gravis and Amyotonia Congenita.**
**May 26,** Dr. B. Abrahams, **Modern Views on Rheumatoid Arthritis.**
**May 27,** Dr. Purves Stewart, **Lesions of the Optic Chiasma.**
**May 28,** Sir Malcolm Morris, **Seborrhœa of the Scalp.**

**THE THROAT HOSPITAL, Golden Square, W.**

**At 5.30 p.m.,**

**May 25,** Mr. C. A. Parker, **Chronic Affections of the Nose (Catarrhal).**
**May 28,** Mr. C. A. Parker, **Chronic Inflammatory Affections of the Nose (non-Catarrhal).**

**THE HOSPITAL FOR SICK CHILDREN, Great Ormond Street, W.C.**

**At 4 p.m.**

**May 28,** Mr. Waugh, **Deformities of the Spine and Limbs.**

The rate of annual subscriptions to The Hospital, either direct from the Office or through agents, is :—
For the United Kingdom... ... ... 15s. a year
To the Colonies and Abroad ... ... 17s. a year
inclusive of postage in each case.

